# Allelic variation in *shrunken2* gene affecting kernel sweetness in exotic-and indigenous-maize inbreds

**DOI:** 10.1371/journal.pone.0274732

**Published:** 2022-09-22

**Authors:** Rashmi Chhabra, Vignesh Muthusamy, Aanchal Baveja, Ashvinkumar Katral, Brijesh Mehta, Rajkumar U. Zunjare, Firoz Hossain

**Affiliations:** 1 ICAR-Indian Agricultural Research Institute, New Delhi, India; 2 ICAR-Indian Grassland and Fodder Research Institute, Jhansi, India; National Bureau of Plant Genetic Resources, INDIA

## Abstract

Sweet corn has become a popular food worldwide. It possesses six-times more sugar than field corn due to the presence of recessive *shrunken2* (*sh2*) gene. Despite availability of diverse sweet corn germplasm, comprehensive characterization of *sh2* has not been undertaken so far. Here, entire *Sh2* gene (7320 bp) among five field corn-(*Sh2Sh2*) and six sweet corn-(*sh2sh2*) inbreds was sequenced. A total of 686 *SNPs* and 372 *InDels* were identified, of which three SNPs differentiated the wild-(*Sh2*) and mutant-(*sh2*) allele. Ten *InDel* markers were developed to assess *sh2* gene-based diversity among 23 sweet corn and 25 field corn lines. Twenty-five alleles and 47 haplotypes of *sh2* were identified among 48 inbreds. Among markers, MGU-*InDel*-2, MGU-*InDel*-3, MGU-*InDel*-5 and MGU-*InDel*-8 had PIC>0.5. Major allele frequency varied from 0.458–0.958. The gene sequence of these maize inbreds was compared with 25 orthologues of monocots. *Sh2* gene possessed 15–18 exons with 6-225bp among maize, while it was 6–21 exons with 30-441bp among orthologues. While intron length across maize genotypes varied between 67-2069bp, the same among orthologues was 57–2713 bp. *Sh2*-encoded AGPase domain was more conserved than NTP transferase domain. Nucleotide and protein sequences of *sh2* in maize and orthologues revealed that rice orthologue was closer to maize than other monocots. The study also provided details of motifs and domains present in *sh2* gene, physicochemical properties and secondary structure of SH2 protein in maize inbreds and orthologues. This study reports detailed characterization and diversity analysis in *sh2* gene of maize and related orthologues in various monocots.

## Introduction

Maize serves as a source of food and nutritional security in the developing countries [1]. Of the various special types, sweet corn has gained popularity as a fresh and processed vegetable [2]. It differs from other maize types in terms of kernel sweetness and possesses higher amount of sugar content [3, 4]. Demand for sweet corn has substantially increased in last few years due to urbanization, increased consumption and availability of organized food processing industries [5, 6]. US$ 1541.72 million and US$ 1475 million worth of import and export were recorded in sweet corn during 2020, respectively [7]. The kernel sweetness is genetically controlled, thus identification of mutations in gene(s) involved in the starch biosynthesis pathway provides gateway to enhance sweetness in kernels [8, 9]. Amongst various mutations reported till date, a recessive *shrunken2* (*sh2*) gene located on chromosome 3L at bin location 3.09, has been widely used in the sweet corn breeding programmes [10]. Recessive *sh2* mutation was first identified as loss of function gene [11] and it was later artificially selected by the breeders to develop sweet corn germplasm globally [12, 13]. Using this *sh2* mutant, large number of *sh2*-based sweet corn inbreds and hybrids has been developed and commercialized [8, 14]. *Sh2* gene encodes the large subunit of *ADP-glucose pyrophosphorylase* (*AGPase*) that catalyzes the conversion of glucose-1-phosphate into ADP-glucose in the starch biosynthesis pathway [15]. Recessive *sh2*-based sweet corn genotypes also known as *‘extra sweet corn’* or *‘super sweet’* accumulate six times higher sugar as compared to ordinary maize at the milky ripe stage [16]. The *sh2*-based varieties have longer shelf life and are better suited for extended storage. This makes the *sh2*-based sweet corn more popular over other types especially in the Asian countries [14]. Though the *sh2* gene in maize was cloned in 1990s [17], limited information on functional and structural diversity of *sh2* gene among diverse maize inbreds is available. This is primarily due to the fact that breeding for sweet corn is limited to a few countries as compared to field corn (used for dried grain purpose) that is grown in more than 180 countries worldwide [18]. In South-East Asian countries, *sh2*-based sweet corn has become very popular in recent times and several composites and hybrids are now available for commercial cultivation [19, 20]. Diverse sweet corn germplasm has been developed by selective introgression of *sh2* allele from donor sources. Two major versions of mutant alleles viz., (i) *sh2-Reference* (*sh2-Ref*) [13] and (ii) *sh2-i* (*sh2*-*intermediate*) created by Dr. Gyula Fiscor [21] have been reported in maize. Recessive *sh2-Ref* allele due to complete loss of function causes extreme crumbling of grains upon maturity [12], while *sh2-i* allele having partial loss of function causes much lesser shrunken phenotype [22]. Of these, *sh2-Ref* has been mainly used in the sweet corn cultivar development worldwide [8]. Manicacci et al. [23] analyzed the variation in partial gene sequence (coding regions: 1228 bp) of complete wild type *Sh2* allele (7320 bp) among a set of 50 maize and teosinte accessions. However, diversity of entire sequence of *sh2*-*Ref* allele (including 5’UTR, coding, non-coding and 3’UTR of >7000 bp) of diverse sweet corn inbreds has not been analyzed and compared with the different wild type (*Sh2*) alleles present in the traditional inbreds. Though various studies on diversity of sweet corn inbreds using genome-wide SSRs have been conducted [24, 25], no effort has been directed to understand the nucleotide diversity in the entire *sh2* gene affecting kernel sweetness in maize. Further, comparative analysis of *sh2* gene of maize with its orthologues among related monocots is also not available in the public domain. The present study was therefore targeted to (i) characterize sequence of *sh2* gene in diverse mutant and wild-type inbreds, (ii) identify haplotypes of *sh2* gene using gene-based *InDel* markers among diverse inbreds, and (iii) study evolutionary relationship of maize *sh2* gene with its orthologues from related monocots.

## Materials and methods

### Genetic material

Five diverse inbreds of wild- (*Sh2*-Wild1 to *Sh2*-Wild5), and six shrunken-type (*sh2-*mutant1 to *sh2-*mutant6) were selected to characterize the wild- (*Sh2*: *first alphabet in capital letter*) and mutant (*sh2*: *first alphabet in small letter*) alleles present among the inbreds (S1 Table). Forty-eight genotypes including 23 sweet corn inbreds with recessive *sh2-Ref* (hereafter *sh2*) and 25 diverse wild-type lines (*Sh2* allele) were used to study the diversity in the *Sh2* gene using gene-based markers. These diverse inbreds have been developed by various breeding centres of India as well as by CIMMYT, Mexico (S2 Table).

### Genomic DNA isolation, amplification and sequencing of s*h2* gene in maize

Five wild- and six shrunken-type inbreds were used for characterization of *sh2* gene. Genomic DNA was isolated from seeds of inbreds employing modified sodium dodecyl sulphate (SDS) extraction protocol [26]. Thirteen overlapping primers were designed with the help of Primer3 Online software covering 7320 bp of *Sh2* gene (GenBank accession no. M81603: wild type allele of *Sh2*; hereafter *Sh2*-Wild-M81603) which amplify the specific fragments ranging from 500-900bp in selected wild and shrunken-type inbreds. These primers were custom-synthesized from M/S Sequencher Pvt. Ltd. PCR was carried out on Veriti 96 well thermal cycler (M/s. Applied Biosystems) in a 50μl reaction in triplicates that consisted of 100ng template DNA, 1x OnePCR^™^ Mix (GeneDireX Ready-to-use PCR master mix) and 0.4 μM of each forward and reverse primer. The PCR conditions were as follows: initial denaturation at 95°C for 5 min, 35 cycles consisting of denaturation at 95°C for 45 s, primer annealing at 60°C for 45 s, primer extension at 72°C for 1 min, and final extension at 72°C for 5 min. Ten microlitre of the amplicon was checked on 2.0% Seakem LE agarose gel and remaining was processed for sequencing from M/s. Sequencher Pvt. Ltd.

### Sequence alignment of gene in selected *sh2*- and wild type maize inbreds

The sequencing results were evaluated using BioEdit and MEGA tool using ClustalW alignment to study variation in *SNPs* or *InDels* among wild (*Sh2*) and mutant (*sh2*) inbreds [27, 28]. The alignment file was then subjected to DnaSP6 software version 6.11.01 to calculate the number of SNPs, *InDels*, haplotypes, number of polymorphic sites, haplotype gene diversity and nucleotide diversity [29]. Putative SNPs which clearly differentiated the wild (*Sh2*) and mutant (*sh2*) allele, were then analyzed using bioinformatics tools viz., SOFTBERRY’s FGENESH and RegRNA2.0 for their functionality [30, 31].

### Analysis of gene diversity in *sh2*- and wild-type maize inbreds

**Fig 1:**
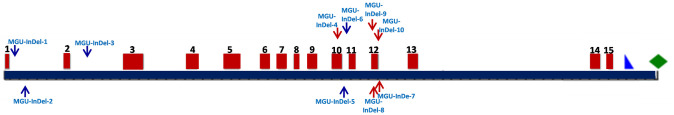
Pictorial representation of primer positions in *Sh2* gene (Blue and red arrows represent positions at intron and exon, respectively; Grey boxes represent exons; blue triangle: Last exon; Green: PolyA tail).

Based on complete sequence of *Sh2* gene in 11 selected genotypes (five wild-type and six mutants), identified *InDel* polymorphisms were considered for further marker development (Fig 1, Table 1). The PCR reactions with *InDel* markers were carried out on standard PCR conditions and annealing temperature for each primer pair was optimized according to the T_m_. The PCR products were run on 4% metaphor agarose and 8% polyacrylamide gel electrophoresis (PAGE) depending upon the size of the *InDel*. The gel profile was analysed in DARwin v6.0 to calculate dissimilarity matrix using Jaccard’s coefficient, and principal coordinate analysis (PCoA) was performed to estimate diversity of *sh2* gene [32]. Dendrogram was constructed from Newick tree employing iTOL (Interactive tree of life) online software [33]. Total number of alleles, major allele frequency, gene diversity, polymorphism information content (PIC) and heterozygosity were also calculated using PowerMarker v3.25 [34].

**Table 1:** Details of markers used in *Sh2* gene diversity analysis.

S. No.	Marker	Sequence (5’→3’)	Amplicon size (bp)	Region
1	MGU-InDel-1	F-CATTTTAGTGGCACGCAATTT R-TTACTTCCATAAAACTGGACATATGAA	100	Intron 1
2	MGU-InDel-2	F-TAGTGGCACGCAATTTTGTC R-TGGACATATGAAGAATGCACCT	80	Intron 1
3	MGU-InDel-3	F-TGCAACTCTTAGAACGCTCA R-CTGCAAATATGGGGCCTAGA	120	Intron 2
4	MGU-InDel-4	F-TCCTCCCAAGAGCTGTACTAGA R-CAACTTTTTGACATGAAGAATTTACAG	98	Exon 10
5	MGU-InDel-5	F-TGTAAATTCTTCATGTCAAAAAGTTGT R-TCCAACATCCTCCCAATAGC	160	Intron 10
6	MGU-InDel-6	F-TGTAAATTCTTCATGTCAAAAAGTTGT R-TGCATGCCTAACACAAATACG	129	Intron 10
7	MGU-InDel-7	F-CGATCCAAAAACACCTTTCTTC R-AAGACATATACCTTGCACTTGTCC	78	Exon 12
8	MGU-InDel-8	F-TTGGAGTCTGCTCACGTGTC R-GGCTACCCTAAATTGCTTGG	157	Exon 12
9	MGU-InDel-9	F-TTGAAATCTAACAAAACAAAAGTCAAA R-GAAGCAATAAAACAATAATTAAATGGA	120	Exon 12
10	MGU-InDel-10	F-AGCTTATGTGGTATCCTCTTGC R-GCCCAGAAAGAATGTATTTCCA	150	Exon 12

### Phylogenetic analysis of *sh2* orthologues in selected monocots

Orthologues of *Sh2* were searched and the sequences were retrieved in related monocots viz., *Oryza sativa* var. *japonica*, *O*. *sativa* var. *indica*, *Brachypodium distachyon*, *Setaria italica*, *Sorghum bicolor*, *Triticum aestivum*, *Hordeum vulgare* and *Aegilops tauschii* across Ensembl Plants’ database using the protein BLAST (BLASTp) tool with an expectation value (e-value) ≤1e^-5^ [35]. A total of 37 sequences, viz., 11 generated in the present study for *Z*. *mays*, one *Sh2*-Wild-M81603 sequence of *Z*. *mays* available in the public domain and 25 orthologous sequences, were used for phylogenetic study (S3 Table). Phylogenetic analysis was performed on the basis of nucleotide and protein sequences using MEGA7 software.

### Gene structure and promoter prediction of *sh2* gene

To predict 5’UTR, transcription start site (TSS), intron-exon boundaries and polyA tail, maize *sh2* gene of the selected inbreds and othologues was then submitted to Softberry’s gene annotation tool (FGENESH). It was based on Hidden Markov Model (HMM) and promoter prediction was also performed with Neural network tool on BDGP (Berkeley Drosophila Genome Project) with the minimum promoter score of 0.8 [36].

### Homology modelling of SH2 protein

To build homology models of protein at different complex levels, a web-based integrated service, SWISS-MODEL was employed [37]. Various parameters, viz., GMQE (Global Model Quality Estimation), QSQE (Quaternary Structure Quality Estimate), Oligomeric state and QMEAN were studied to build a putative model for the protein under investigation. Protein data bank (PDB) file of the selected model was then submitted to RAMPAGE server for Ramachandran Plot assessment [38]. It shows statistical distribution of backbone dihedral angles ϕ and ψ in different combinations [39].

### Domains analysis and physicochemical properties of SH2 protein

Protein domains and features in SH2 protein (large sub-unit of AGPase) were identified from Ensembl Plants’ database. These domains were annotated by seven major protein signature databases [PANTHER, CDD (Conserved domain database), Gene3D, PROSITE, Superfamily, Pfam and TIGRFAM through InterPro]. Further, in selected inbreds and orthologues, protein domain search was carried out with MOTIF Search tool. The different physicochemical properties of SH2, viz., molecular weight, aliphatic index (AI), isoelectric point, Grand average of hydropathy (GRAVY) and instability index were estimated for primary structure of all the retrieved and predicted proteins using ProtParam tool of ExPASy and PEPSTATS tools [40, 41].

## Results

### Sequence characterization of *sh2* gene in selected mutant and wild-type inbreds of maize

**Fig 2:**
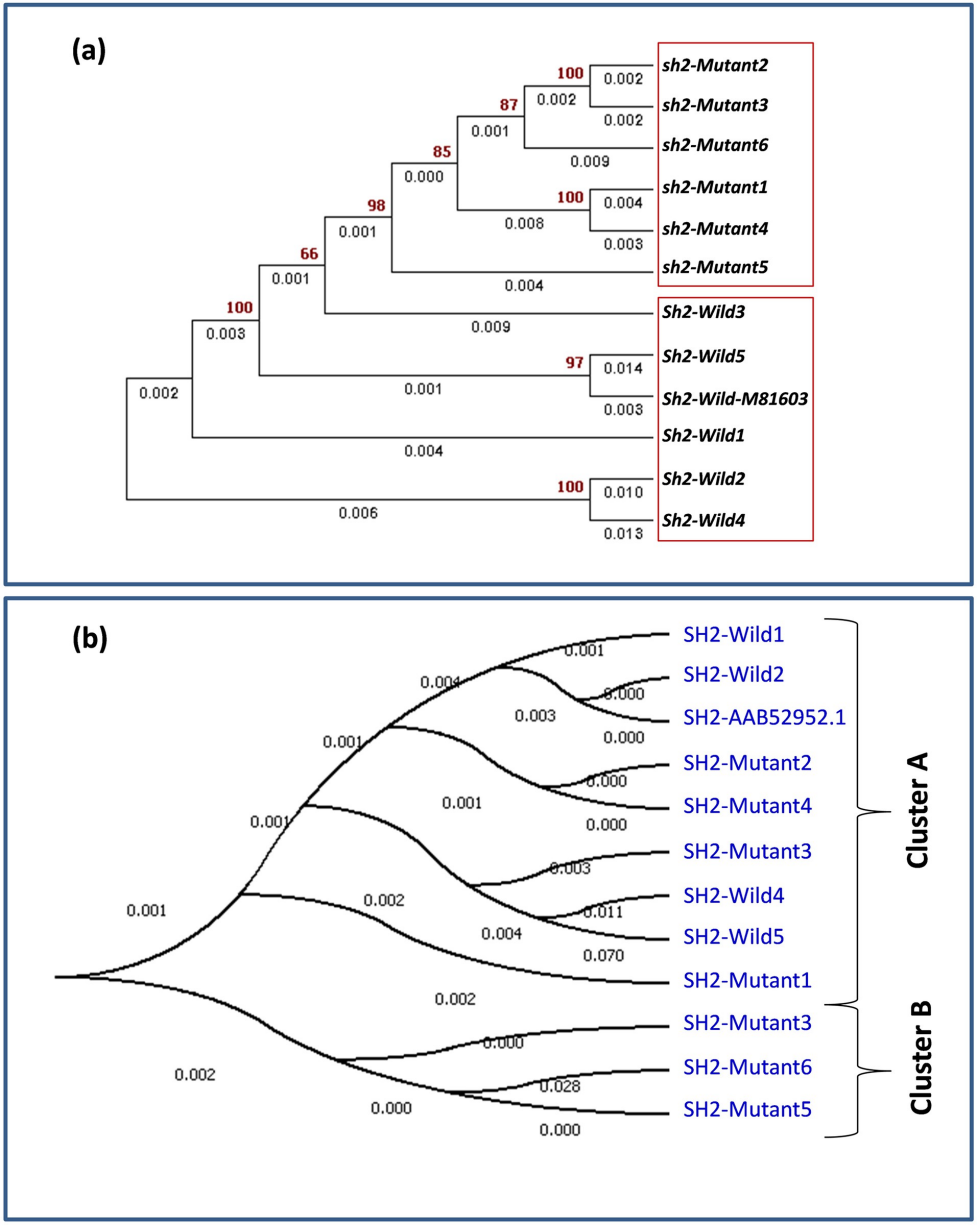
Phylogenetic analysis of selected *sh2-*mutants and wild inbreds on the basis of (a) nucleotide sequences and (b) protein sequences.

Sequence analysis of entire *sh2* gene among 11 selected inbreds (five wild type and six mutants) along with the *Sh2*-Wild-M81603 revealed a total of 686 *SNPs* and 372 *InDels*. Parsimony informative sites and singleton variable sites were 253 and 323, respectively. Number of identified haplotypes was 12 with a haplotype gene diversity of 1.000, while standard deviation of haplotype diversity and variance were 0.034 and 0.00116, respectively. Tajima’s D value was -0.94172 (statistically non-significant, P>0.10) with nucleotide diversity (Pi) of -0.94172 and theta (per site) from Eta of 0.02874. Average length of *InDel* was 6.004 bp with *InDel* diversity (*ki*) of 23.879. Forty-six conserved regions were observed with p-value ranging from 0.0000–0.0426. Tajima’s D-values for two populations of mutant and wild were calculated to be -1.30991 and -0.40253, respectively, which were not statistically significant at P>0.10. Among all polymorphic sites, only seven SNPs clearly differentiated the mutant and wild-type allele, viz., two (SNP583 and SNP755) in 5’UTR and five (SNP5112, SNP5228, SNP5379, SNP6226 and SNP6446) in intronic regions, but only three of these (SNP583, SNP755 and SNP5112) were exploited to develop markers [8]. Cluster diagram employing Neighbour-Joining method with a total branch length of 0.101 revealed diverse relationships among mutants (*sh2*) and wild-type (*Sh2*) inbreds (Fig 2a). Among the inbreds, (i) *sh2-*mutant2 and *sh2-*mutant3, and (ii) *sh2-*mutant1 and *sh2-*mutant4 were clustered together, while among the wild type inbreds, (i) *Sh2*-Wild2 and *Sh2*-Wild4, and (ii) *Sh2*-Wild-M81603 and *Sh2*-Wild5 were together. The significance of identified polymorphisms in coding sequences viz., non-synonymous (Ka)/synonymous (Ks) nucleotide diversity ratio was estimated for *sh2* gene in selected maize genotypes. Among the selected 11 maize inbreds, *sh2-*mutant1 and *sh2-*mutant4 had the minimum Ka/Ks ratio, which indicated very less non-synonymous changes, therefore having less deviation from evolutionary point of view. *Sh2*-Wild3 had the highest Ka/Ks ratio followed by *Sh2*-Wild4, hence they were more significantly diverse suggesting that particular allele is under selective pressure (S4 Table).

### Diversity and haplotype analysis of *sh2* gene among diverse maize inbreds using *InDel* markers

**Fig 3:**
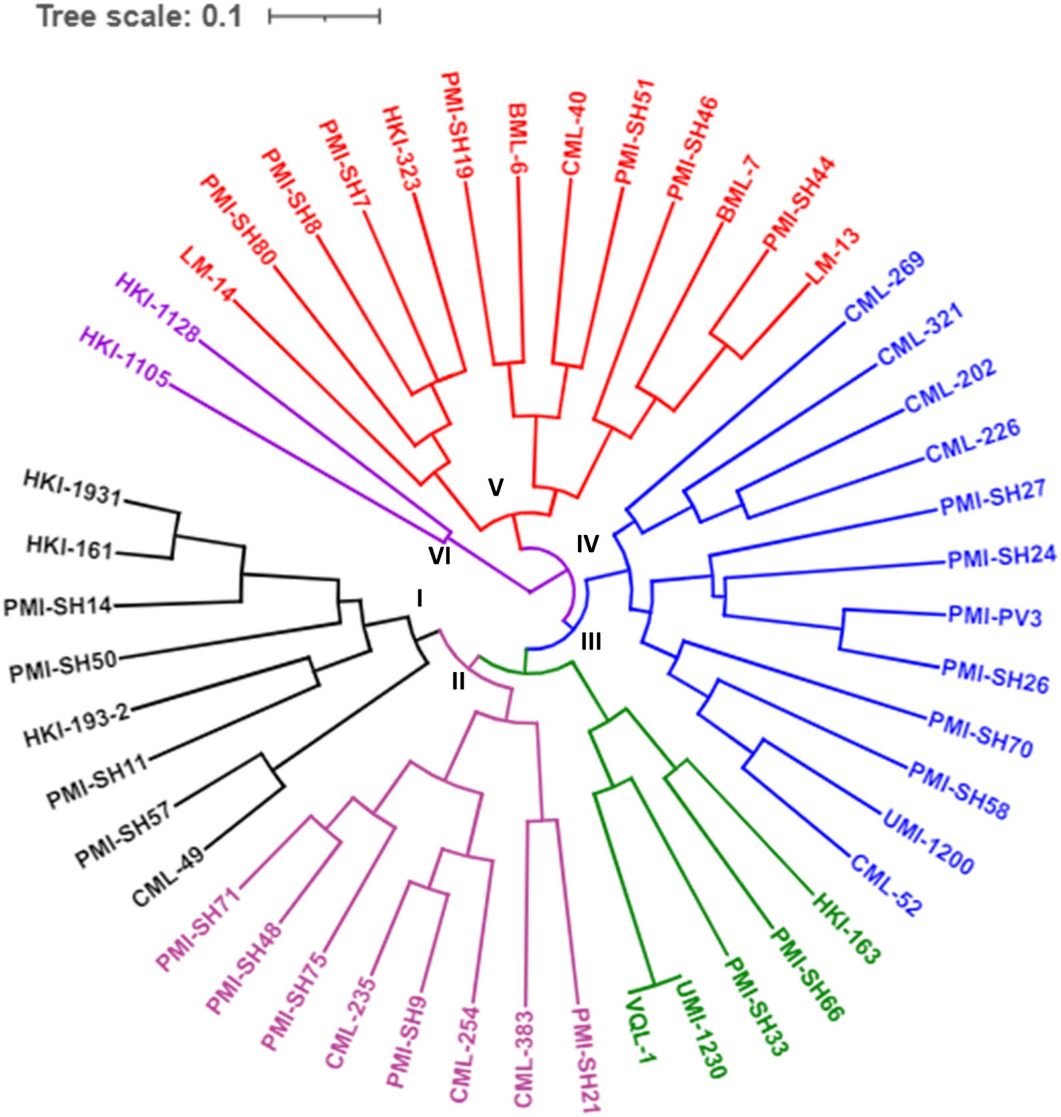
Dendrogram representing *sh2*-gene based diversity using *InDel* markers.

**Fig 4:**
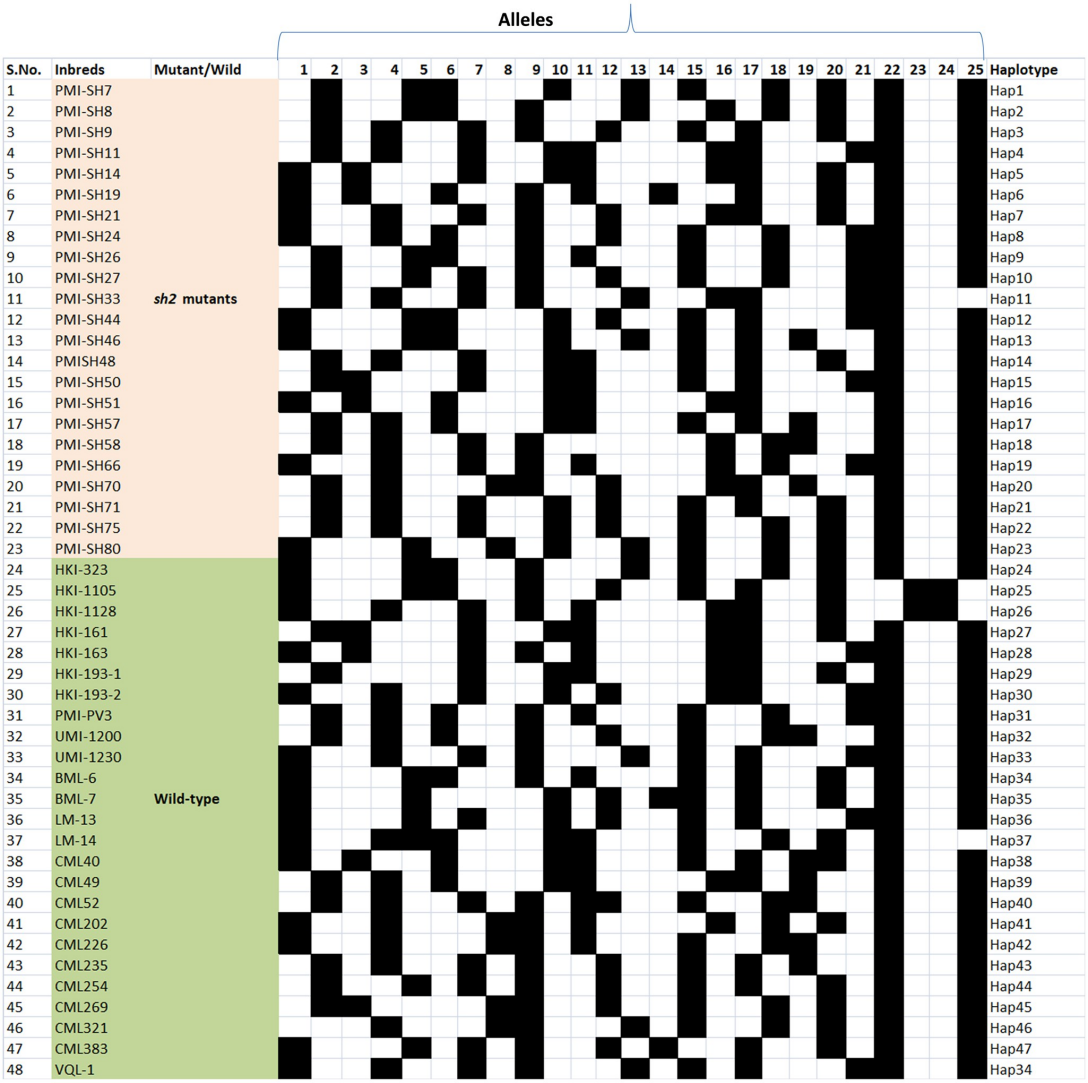
Haplotype of *Sh2* alleles using 10 markers; each row represents the selected mutant and wild genotypes and columns represent the allele for a given marker, black box colour: Presence of DNA band, white box colour: Absence of DNA band.

*Sh2*-sequence comparison among the wild and mutant inbreds revealed presence of 372 *InDels*. Ten *InDels* of >2bp were exploited for gene-based marker diversity using 48 diverse genotypes (Table 1). All the *InDel* markers (MGU-*InDel*-1 to MGU-*InDel*-10) were polymorphic among the diverse genotypes. A total of 25 alleles were generated with allelic range of 1 to 3 per locus (Table 2). Major allele frequency ranged from 0.458 (MGU-*InDel*-5) to 0.958 (MGU-*InDel*-9) with mean of 0.6227. Mean PIC and gene diversity were 0.38 and 0.46, respectively. No heterozygosity was found in the panel of inbreds using these gene-based markers (Table 2). Genetic dissimilarity ranged from 0 to 0.94 with an average of 0.54. Genetic dissimilarity grouped the diverse genotypes into six major clusters. Cluster-V comprised of the highest number of inbreds (13 inbreds) followed by cluster-IV with 12 inbreds, while cluster-VI comprised of only two genotypes (HKI-1128 and HKI-1105) (Fig 3). Further, marker scores were exploited for haplotype study among 48 inbreds. In total, 47 haplotypes were observed with VQL-1 and UMI-1230 possessing the same haplotype. All the 23 sweet corn inbreds having recessive *sh2* allele had different haplotypes as depicted by *InDel* markers. A pictorial representation of haplotypes in respective genotypes is presented in Fig 4.

**Table 2:** Summary statistics of genotyping assay of 48 inbred lines.

S. No.	Marker	Major allele frequency	No. of alleles	Gene diversity	Heterozygosity	PIC
1	MGU-InDel-1	0.5000	2.0000	0.5000	0.0000	0.3750
2	MGU-InDel-2	0.5417	3.0000	0.5938	0.0000	0.5228
3	MGU-InDel-3	0.5000	3.0000	0.6033	0.0000	0.5246
4	MGU-InDel-4	0.5833	2.0000	0.4861	0.0000	0.3680
5	MGU-InDel-5	0.4583	3.0000	0.6293	0.0000	0.5531
6	MGU-InDel-6	0.6042	3.0000	0.5200	0.0000	0.4351
7	MGU-InDel-7	0.6250	2.0000	0.4688	0.0000	0.3589
8	MGU-InDel-8	0.5000	3.0000	0.6215	0.0000	0.5499
9	MGU-InDel-9	0.9583	2.0000	0.0799	0.0000	0.0767
10	MGU-InDel-10	0.9565	2.0000	0.0832	0.0000	0.0797
**Mean**		0.6227	2.5000	0.4586	0.0000	0.3844

PIC = Polymorphism information content

### Phylogenetic analysis of *sh2* sequences of maize and its orthologues in monocots

**Fig 5:**
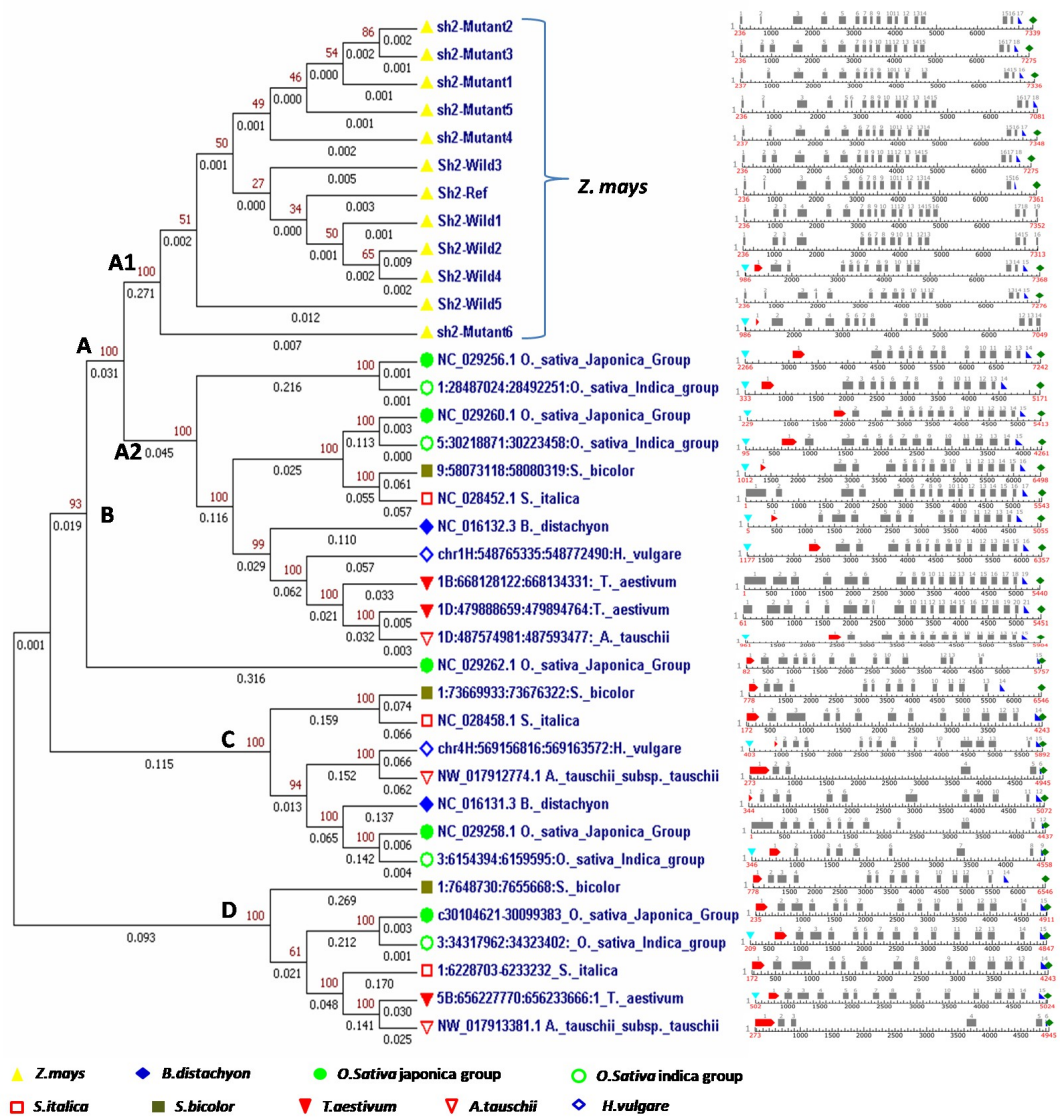
Evolutionary relationship of *Sh2* gene in maize and its orthologues in selected monocots with their gene architecture.

The phylogenetic analysis was performed to understand the evolutionary relationship of *sh2* gene among a total of 37 accessions of *Z*. *mays*, *S*. *bicolour*, *T*. *aestivum*, *A*. *tauschii*, *O*. *sativa* (*indica* and *japonica*), *H*. *vulgare*, *S*. *italica* and *B*. *distachyon*. Eleven sequences of maize generated under the study, and *Sh2*-Wild-M81603 were used for the phylogenetic analysis. Clustering method based on nucleotide separated 37 genotypes (12 maize and 15 orthologues) into four major clusters with the branch length of 3.810 (Fig 5). The percentage of replicate trees ranged from 27–100% in which the associated taxa grouped together with bootstrap test of 10000 replicates. Cluster-A and cluster-B had 23 genotypes and one genotype, respectively. The cluster-A was further divided into two sub-clusters, viz., -A1 with 12 and -A2 with 11 genotypes. Interestingly, all maize sequences were grouped into single cluster-A1. Phylogenetic analysis revealed that the largest cluster-A consisted of 12 members of maize, four members of rice, two members of wheat species, and one each of *A*. *tauschii*, *B*. *distachyon*, *H*. *vulgare* and *S*. *italica*. While, cluster-C comprised of two members of rice, one member each of *A*. *tauschii*, *B*. *distachyon*, *H*. *vulgare*, *S*. *bicolour* and *S*. *italica*. Cluster-D included seven members comprising two of rice, and one each of *A*. *tauschii*, *B*. *distachyon*, *H*. *vulgare*, *S*. *bicolor* and *S*. *italica*. No species-specific cluster could be identified in cluster-B, -C and -D indicating sequence diversity among different accessions of crops for *sh2* locus.

### Gene structure and promoter prediction for *sh2* in maize and its orthologues

Gene structure prediction revealed 15–18 exons among maize sequences, while 6–21 exons were predicted among the orthologues. It also predicted transcription start site (TSS), positions of first, internal and last coding sequences (CDSf, CDSi and CDSl) and PolyA signal sequence. Twenty four of the predicted genes were found to be complete with respect to protein coding sequence as all the genes had CDSf, CDSi and CDSl. The exons and introns boundaries of *sh2* gene in maize genotypes and among the orthologues have been annotated in Fig 5. Length of the introns across the maize inbreds (sequenced in the present study) ranged from a minimum of 67 bp in *sh2-*mutant6 to maximum of 2069 bp in *Sh2*-Wild2. The gene architecture of selected orthologues when compared with 12 maize sequences demonstrated that the minimum (57) and maximum (2713) number of bases in introns were in *A*. *tauschii* (NW_017912774.1). The comparative lengths of exons across the *sh2* sequences of maize varied from 6bp (*Sh2*-Wild2) to 225bp observed in all 11 maize *sh2* sequences generated under the study. *Sh2*-Wild-M81603 possessed 16 exons ranging from 6 to 250bp in length. In case of orthologues, the number of bases in exon varied from 30bp in *A*. *tauschii* (NW_017912774.1) to 441bp in *S*. *italica* (NC_028458.1) (Fig 5). All genes were predicted with a strong promoter having score between 0.9–1.0.

### Characterization of SH2 protein in selected mutant and wild-type inbreds of maize

The comparison of protein sequences of wild and mutant inbreds with reference sequence, SH2-AAB52952.1 (GenBank accession: *Sh2*-Wild-M81603) showed 159 variable regions (amino acid substitutions) and 43 *InDels*. There were 17 *InDels* among the mutant sequences and 26 among the wild sequences, when compared with SH2-AAB52952.1 protein sequence (Table 3). The evolutionary history among predicted SH2 protein in mutant- and wild- type maize genotypes was generated with branch length of 0.13439593 (Fig 2b). Phylogenetic analysis revealed two major clusters viz., -A and–B, each having nine and three inbreds, respectively. Cluster-B possessed all mutant version of *sh2* gene (SH2*-*mutant3, SH2*-*mutant5 and SH2*-*mutant6), while cluster-A had mix of both mutant- and wild- type SH2 proteins.

**Table 3:** Polymorphic data in comparison with *Sh2*-Wild-M81603 coding region and SH2 protein.

S. No.	*Sh2* alleles	Gaps	Coding region		Sequence similarity (%)	No of ORFs	Length of protein (aa)	Protein sequence	
			SNPs	*InDels*				Substitutions	*InDels*
1	*sh2-*mutant1	93 (1.26%)	122	16	966	1	523	9	3
2	*sh2-*mutant2	48 (0.65%)	84	14	983	1	558	9	2
3	*sh2-*mutant3	66 (0.89%)	84	9	981	1	515	3	3
4	*sh2-*mutant4	81 (1.09%)	124	15	972	1	565	3	1
5	*sh2-*mutant5	63 (0.85%)	79	9	979	1	527	5	2
6	*sh2-*mutant6	96 (1.3%)	113	14	974	1	491	37	6
7	*Sh2*-Wild1	60 (0.81%)	70	9	980	1	620	15	5
8	*Sh2*-Wild2	118 (1.75%)	177	13	949	1	487	15	5
9	*Sh2*-Wild3	78 (1.05%)	105	9	976	1	492	8	3
10	*Sh2*-Wild4	73 (0.90%)	206	8	952	1	509	24	5
11	*Sh2*-Wild5	76 (1.00%)	117	10	974	1	467	26	8

### Phylogenetic analysis of SH2 protein sequences of maize and its orthologues

Clustering method based on protein sequences separated 37 genotypes (12 maize and 15 orthologues) into three key clusters viz., cluster -A, -B and -C with twenty three, seven and two genotypes, respectively. The cluster-A can be further separated into two sub-clusters -A1 and -A2 with 14 and nine members, respectively (S1 Fig). Maize SH2 protein was grouped into a single sub-cluster-A1. The clustering pattern also revealed that maize SH2 protein is closer to rice SH2 orthologues compared to other monocots. Cluster-A3 comprised of two accessions of *T*. *aestivum* and one each of *O*. *sativa* var. *japonica*, *O*. *sativa* var. *indica*, *S*. *bicolor*, *H*. *vulgare*, *A*. *tauschii*, *S*. *italica* and *B*. *distachyon*, while cluster-B had seven accessions comprising two accessions of *O*. *sativa* var. *japonica* and one each of *S*. *bicolor*, *A*. *tauschii*, *S*. *italica*, *O*. *sativa* var. *indica* and *T*. *aestivum*. Cluster-C also had seven accessions, one each of *O*. *sativa* var. *japonica*, *O*. *sativa* var. *indica*, *S*. *bicolor*, *H*. *vulgare*, *A*. *tauschii*, *S*. *italica* and *B*. *distachyon*.

### Homology modelling of SH2 protein

**Fig 6:**
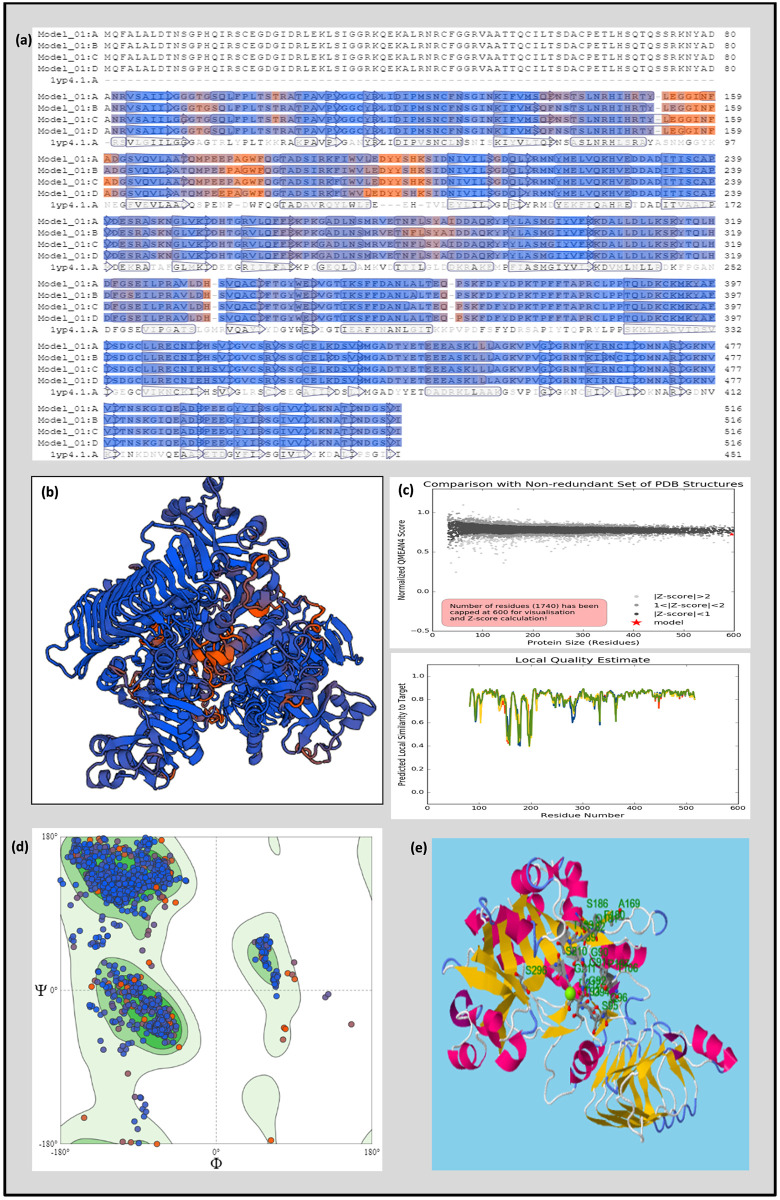
Homology-based protein model of large subunit of AGPase; (a) alignment with similar templates; (b) protein model generated using SWISS-MODEL; (c) comparison with non-redundant set of PDB structures and Local quality estimate; (d) Ramachandran Plot and (e) Ligand-binding sites predicted using RaptorX.

The protein sequence of large subunit of AGPase (SH2) was searched for similar templates. A total of 726 templates were found identical to target sequence having identity between 18.46 and 49.76 (Fig 6a). Although the similarity was not very high, top four templates were selected for modelling having GMQE score between 0.64–0.66, which depicted the expected quality of the model and QSQE score of 0.49–0.52. All those templates were found in homotetramer state and had 2ADP and 1ADQ as ligands. The 1yp4.1.A template (Glucose-1-phosphate adenylyl transferase small subunit) found by HHBlits with an identity score of 49.76% was chosen as the best template for homology modelling for large subunit of AGPase. The Z-score is a factor of model quality which calculates the total energy of the protein structure. The curves obtained in protein-protein interaction fingerprint (PPI fingerprint) provided information of template interface, and its value less than 0 represented that the residues on interface were less vulnerable to mutate than those on surface, which was in agreement with our model. The QMEAN Z-score denoting ‘degree of nativeness’ was -1.17 (Fig 6a–6d).

The assessment of Ramachandran plot of large subunit of AGPase for stereochemical and kinetic properties showed 95% (490) of amino acids were in most favoured zone, 4% (20) in allowed region and 0.6% (3) in outlier region. MolProbity score was 1.4 with a clash score of 1.04. Although AGPase has a well-documented role in starch metabolism, where ATP and glucose-1-phosphate are major substrates, no other substrate/ligand was predicted for developed model. However, online server Raptor-X-binding site prediction determined Mg^+2^ as ligand with pocket multiplicity value of 97 with p-value 1.75e^-13^ on amino acid residues viz., L89, G90, G91, G92, T93, G94, S95, Q96, T106, P107, A169, F180, Q181, G182, T183, S186, S210, G211, D212 and S296, depicting that the predicted pocket is true (Fig 6e).

### Domains, motifs and features of SH2 protein

The SH2-AAB52952.1 protein was annotated with 14 domains with three major domains including (i) AGPase domains (1, 2 and 3); (ii) nucleotide triphosphate transferase (NTP) and (iii) bacterial transferase hexapeptide domains (Table 4, S2a and S2b Fig). In most of the *sh2* mutants, AGPase domain was intact as in SH2-AAB52952.1 sequence, but NTP transferase domain was distorted or short in length in SH2*-*mutant5 and SH2*-*mutant6 (S5 Table). Among the orthologues, all the accessions possessed NTP transferase domain except seven accessions, viz., Q10Q61 (*O*. *sativa* var. *japonica*), I1H8B7 (*B*. *distachyon*), A0A287P195 (*H*. *vulgare*), C5WTQ1 (*S*. *bicolor*), B8AQHO (*O*. *sativa* var. *indica*), K4AAH8 (*S*. *italica*) and M8BCF1 (*A*. *tauschii*) which had only AGPase domain. Besides, these three domains, some of the orthologues also possessed beta-helix, fucokinase, CTP transferase, DUF4889, NHR2 and DUF4954 domains.

**Table 4:** Domains and features of SH2 protein in *Zea mays*.

S. No	Domain source	Start	End	Description	Accession
1	PANTHER	1	516	-	PTHR43523
2	PANTHER	1	516	-	PTHR43523:SF1
3	CDD	86	347	-	cd02508
4	Gene3D	382	516	-	21601010
5	CDD	384	510	-	cd04651
6	PROSITE patterns	90	109	ADP-glucose pyrophosphorylase, conserved site	PS00808
7	PROSITE patterns	179	187	ADP-glucose pyrophosphorylase, conserved site	PS00809
8	PROSITE patterns	295	305	ADP-glucose pyrophosphorylase, conserved site	PS00810
9	TIGRFAM	85	481	Glucose-1-phosphate adenylyltransferase	TIGR02091
10	Gene3D	66	381	Nucleotide-diphospho-sugar transferases	39055010
11	Superfamily	84	478	Nucleotide-diphospho-sugar transferases	SSF53448
12	Pfam	86	362	Nucleotidyltransferase domain	PF00483
13	Superfamily	380	516	TrimericLpxA-like superfamily	SSF51161

### Physicochemical properties of SH2 protein in diverse maize inbreds and its orthologues

The coding sequence of SH2 protein in maize was 1908 bp long with a translated protein of 516 amino acids. Protparam and PEPSTATS analyses of the SH2-AAB52952.1 protein revealed that it consisted of 60 negatively charged and 55 positively charged amino acids. Aliphatic index and GRAVY were found to be 85.06 and -0.207, respectively, which indicated the non-polar nature of SH2 protein (Table 5). The instability index was computed to be 38.88, which classified the protein as stable. The predicted secondary structure had 138 α-helices, 26 β-turns, 238 random coils and 114 extended strands (S3a Fig). The SH2 protein was predicted with four unfolded regions with a total of 53 amino acids out of 516 amino acids, and the longest unfolded region had 26 residues (S3b Fig).

**Table 5:** Physiochemical properties of SH2 protein and orthologous protein.

S. No.	Sequence Name	Genus	No of amino acids	Molecular weight	Isoelectric point	Negatively charged (Asp+Glu)	Positively charged (Arg+Lys)	Instability index	Aliphatic index	GRAVY
1	SH2*-*mutant1	*Z*. *mays*	534	58725.89	631	60	56	35.46 (stable)	88.20	-0.167
2	SH2*-*mutant2		558	61604.51	759	61	62	36.22 (stable)	86.86	-0.174
3	SH2*-*mutant3		515	56709.83	810	56	59	37.81 (stable)	82.39	-0.221
4	SH2*-*mutant4		565	62538.40	723	63	63	37.17 (stable)	85.95	-0.202
5	SH2*-*mutant5		527	57803.05	683	60	59	35.76 (stable)	85.31	-0.19
6	SH2*-*mutant6		491	54568.32	683	52	51	42.10 (unstable)	82.42	-0.185
7	SH2-Wild1		620	68833.88	838	63	69	39.45 (stable)	85.26	-0.164
8	SH2-Wild2		487	54402.43	791	55	57	35.87 (stable)	85.73	-0.194
9	SH2-Wild3		492	54562.15	615	57	52	38.21 (stable)	85.45	-0.213
10	SH2-Wild4		509	56670.07	621	63	58	38.01 (stable)	85.05	-0.156
11	SH2-Wild5		467	51347.98	831	50	54	35.47 (stable)	87.73	-0.172
12	SH2-AAB52952.1		516	57071.02	616	60	55	38.88 (stable)	85.06	-0.207
13	Q688T8	*O*. *sativa* var. *japonica*	519	57653.73	634	63	59	35.72 (stable)	82.85	-0.212
14	Q7G065		518	57574.64	548	66	53	38.17 (stable)	82.28	-0.221
15	Q0D7I3		509	55829.02	792	57	59	40.25 (unstable)	84.73	-0.107
16	Q10Q61		415	45754.59	628	46	41	41.73 (unstable)	100.51	-0.041
17	Q6AVT2		511	55427.08	701	60	60	35.55 (stable)	88.26	-0.129
18	I1H8B7	*B*. *distachyon*	415	45653.59	664	44	42	44.44 (unstable)	99.86	-0.045
19	I1HFZ1		522	57901.08	606	65	59	40.50 (unstable)	85.19	-0.153
20	A0A287PI95	*H*. *vulgare*	504	55242.17	837	51	54	49.25 (unstable)	91.92	-0.224
21	C3W8L1		523	57932.92	617	65	60	43.13 (unstable)	80.75	-0.242
22	C5WTQ1	*S*. *bicolor*	415	45792.64	647	46	43	40.79 (unstable)	99.08	-0.066
23	C5WLV9		507	55381.18	833	59	62	37.03 (stable)	89.86	-0.132
24	A0A1Z5R3X9		679	75012.66	906	68	83	45.89 (unstable)	78.57	-0.223
25	B8AR31	*O*. *sativa* var. *indica*	508	55153.73	701	60	60	35.46 (stable)	88.39	-0.14
26	A2Y7W1		519	57653.73	634	63	59	35.72 (stable)	82.85	-0.212
27	P93430		518	57574.64	548	66	53	38.17 (stable)	82.28	-0.221
28	B8AQH0		362	39849.69	591	42	33	40.18 (unstable)	101.24	-0.015
29	K4AAH8	*S*. *italica*	415	45730.61	632	46	42	40.84 (unstable)	100.96	-0.038
30	K4A7B2		605	66247.49	929	67	81	45.58 (unstable)	83.54	-0.306
31	K3Z5F3		518	57509.39	612	62	57	39.41 (stable)	80.73	-0.229
32	A5GZ74	*T*. *aestivum*	503	54774.44	831	58	61	33.32 (stable)	87.69	-0.133
33	Q7XJA9		522	57706.51	589	66	59	43.58 (unstable)	79.58	-0.27
34	P12299		522	57808.72	612	65	60	42.86 (unstable)	80.52	-0.253
35	M8AY46	*A*. *tauschii*	478	52231.75	800	53	55	30.83 (stable)	91.03	-0.040
36	A0A0U4H004		522	57808.72	612	65	60	42.86 (unstable)	80.52	-0.253
37	M8BCF1		436	48124.69	527	63	50	39.60 (stable)	93.94	-0.259

When SH2 protein in maize genotypes was compared with each other, and it was found that leucine is predominant followed by either serine or glycine in all wild genotypes with a range of 41–56% leucine, but in case of SH2-mutants, glycine was the predominant amino acid except SH2-mutant2 (leucine was maximum with 46%) and SH2-mutant4 (serine was predominant with 47%). In SH2-AAB52952.1, serine and leucine were equally predominant followed by glycine and alanine. Among the selected genotypes of maize, SH2-mutant1 had instability index of 42.10 depicting its unstable nature. The aliphatic index among the 12 genotypes of maize ranged from 82.39–87.73, which was comparable to SH2-AAB52952.1 protein, depicting its thermostable soluble nature, while negative GRAVY value revealed the non-polar nature of the peptide.

The sequence of SH2 protein in selected maize genotypes, when compared to orthologues of selected monocots, revealed that out of selected five accessions of *O*. *sativa* var. *japonica*, three (Q688T8, Q7G065 and Q6AVT2) were stable and two (Q0D713 and Q10Q61) were unstable proteins. Proteins of *B*. *distachyon* and *H*. *vulgare* were stable, whereas out of three orthologues of *S*. *bicolor*, two (C5WTQ1 and A0A1Z5R3X9) were unstable with instability index of 40.79 and 45.89, respectively. In case of *O*. *sativa var*. *indica*, out of four selected orthologues, only one (K4AAH8) was unstable with instability index of 40.18, whereas out of three orthologues of *T*. *aestivum*, only (A5GZ74) one was highly stable with the value of 33.32. Among the three orthologues of *A*. *tauschii*, one accession (M8AY46) was highly stable, one M8BCF1 (39.60) was moderately stable, while A0A0U4H00 (42.86) was unstable. Aliphatic index of selected protein sequences ranged from 78.57 (A0A1Z5R3X9) in *S*. *bicolor*, which suggested its lower solubility and it went upto 101.24 (B8AQH0) in *O*. *sativa* var. *indica*, showing increased solubility of this protein, as compared to other orthologues (Table 3). The SOPMA comparison of selected maize sequences and *sh2* orthologues showed that the percentage of α-helices in those proteins ranged from 21.96–33.26%; with minimum in SH2-mutant1 and maximum in SH2-Wild2 sequence of maize, whereas that of extended strands varied from 19.5–27.07%. The percentage of β-turns ranged from 4.79–8.45% and that of random coils varied from 33.7–50.39%.

## Discussion

Sweet corn generates livelihood to millions of farmers due to its diverse usage worldwide [8, 10, 20]. In maize, several recessive genes like *sh2*, *sugary1* (*su1*) and *sugary enhancer1* (*se1*) enhance kernel sweetness, and often used alone or in combination for the development of sweet corn hybrids [42]. Of these, *sh2*-based sweet corn is more popular in Asian countries as it possesses three-time more sweetness besides having better shelf-life after harvest over *su1* types [16, 20]. The large subunit of AGPase is encoded by *Sh2* gene [43]. Mutation in large subunit caused noteworthy decrease in endosperm’s AGPase activity and starch content. Of these, *sh2* mutant has been abundantly used in sweet corn cultivar development across countries [44]. *Sh2* gene has been reported to have low nucleotide diversity and highly conserved protein sequence among crops [23]. In most of the plants, AGPase is a tetrameric protein complex with two large and two small subunits [45]. However, various mutations have been reported in large subunit of AGPase encoded by *Sh2*. In maize, *SNPs*, *InDels* and chromosomal rearrangements in *sh2 gene* have been reported [12, 46–48]. Tuncel et al. [49] reported that mutation in *OsAGPL2* gene in rice had severely shrivelled seeds. AGPase activity in endosperm of 16 barley mutants had 15–25% of that observed in wild-type [50]. Thus, loss or impaired functions in various mutants of *sh2* cause elevated kernel sweetness in the endosperm [10]. In the present investigation, comprehensive characterization of *sh2* gene in a diverse set of mutant- and wild- type inbreds of maize has been undertaken and compared with the orthologous sequences of the related monocot species.

### Diversity analysis in *sh2* gene among exotic and indigenous maize inbreds

Gene-based *InDel* markers showed a low degree of polymorphism among the selected genotypes with an allele frequency of an average of 2.5 alleles per locus. However, the allele frequency is much lower than that observed in a microsatellite-based diversity analysis performed among sweet corn genotypes [24, 25, 51] and Zheng et al. [52] reported 3.26–5.20 alleles/locus. Babu et al. [53] reported higher number of alleles/locus using SSRs, as compared to that employing lysine- and tryptophan-biosynthesis pathway-specific candidate gene-based SSR in a set of quality protein maize (QPM) inbreds. These SSRs are present mostly in non-genic region, and high level of allelic polymorphism is due to reasons like recombination errors, unequal crossing over and replication slippage at the SSR locus [54]. On the other hand, high degree of mutations within a gene is not tolerated as functional errors that may lead to loss of the genotype from the populations. Average PIC among the 48 genotypes was also low compared to the earlier studies dealt with genome-wide SSRs [24, 25, 50, 52]. This is in accordance with the study carried out by Manicacci et al. [23] where low degree of nucleotide polymorphisms was observed in coding region of *sh2* gene across 50 accessions of maize and teosinte. Further, absence of heterozygosity in *sh2* gene depicted the complete homozygous nature of the inbreds. This was possibly due to stringent maintenance of the inbreds due to their strict self-pollination for many generations. However, some of the genome-wide SSR loci distributed throughout the genome earlier revealed some degree of heterozygosity among the sweet corn inbreds [25].

With the advancement of sequencing techniques, nowadays it is possible to generate haplotype information for identifying unknown germplasm [55]. Haplotypic structural variability strongly affects the frequency and distribution of recombination events in maize. Beyond allele frequencies, haplotype data collected in population samples contain information about the history of allelic associations in gene genealogies, and this is of tremendous potential for conservation genomics. In a study carried out by Yao and Schnable [56], *cis*-effects were identified that examined up to 3-fold differences in recombination rates across the *a1-sh2* interval among the different haplotypes in maize and teosinte due to several large *InDel* polymorphisms in teosinte relative to maize. This permits the dissection of variation in introgression rates across the genome, thus unfolding the evolutionary processes [55]. Retrieving information about haplotypes within populations considerably improves the estimation of various factors relevant to evolutionary conservation. In the present study, *InDel*-based markers were used to identify 47 haplotypes of *sh2* gene in a diverse set of maize inbreds of exotic and indigenous origin. Two inbreds viz., VQL-1 and UMI-1230 showed similar haplotype thereby suggesting the origin of *Sh2* gene from a common ancestry. *InDel* markers are co-dominant and involves simple and easy PCR assay, and requires standard gel electrophoresis for separation of amplicons. Further, *InDel*-based assay does not involve high cost as required in sequence-based approach, as shown in similar studies carried out on *su1* and *fatb* genes for allelic diversity and comprehensive molecular characterization [2, 57]. Our study showed that *Sh2*-specific *InDel* markers developed in the present study would be useful in identifying haplotypes of unknown lines to understand their lineage.

### Molecular characterization of *sh2* gene and large subunit of AGPase

Upon characterization of *sh2* gene in diverse mutant- and wild-type inbreds, it was observed that each genotype possessed different allele, thereby showing highly diverse nature of selected eleven lines. Most of the identified *SNPs* and *InDels* were present in either 5’UTR or intronic region. *Sh2* gene in both mutant-and wild- type maize inbreds had longer introns. However, the dissimilarity of all eleven sequences with *Sh2-*M81603 (GenBank Accession) was found to be very low (1.7 to 5.1%). Manicacci et al [23] studied molecular evolution of *Sh2* gene in maize by comparing coding region (4669 bp) on different accessions of maize and teosinte. They also found very less nucleotide variations suggesting a purifying selection effect in whole species predating domestication. In the present study as well, majority of SNPs and *InDels* were in the non-coding regions thereby suggesting the conserved functional nature of the *sh2* gene in maize. We, earlier have developed, SNP-based allele-specific markers surrounding these three SNPs for marker-assisted selection of *sh2* gene in sweet corn breeding programme [8].

The 3D structure of proteins is important to study protein dynamics, function, as well as protein-ligands interaction. If we look upon the structure of large subunit of AGPase in eukaryotes, the protein is a tetramer comprising two separate subunits having catalytic and modulatory roles. Modulatory subunit is related to changes in enzyme regulation in different plant tissues. Prediction of the AGPase secondary structure suggests similar folding pattern as in other sugar nucleotide pyrophosphorylases, revealing that they evolved from the same antecedent [58]. The atomic resolution structure of AGPase was first proposed by Jin et al. [59] which provided strong understandings for the mechanism of enzyme catalysis and its allosteric regulation. However, the 3D structure of large subunit of AGPase in maize is not available in public domain. Therefore, homology modelling was performed as a practical option to decipher the 3D model for the same. The statistics of residues in favoured and allowed region and a very low percentage in the outlier region suggested that the Ramachandran plot for AGPase is acceptable. Among the orthologues, the average of pI was 7.12 (5.27–9.29) depicting that the enzyme probably precipitates in basic buffers. Though in some of the orthologues, the pI is very much less than 7, *viz*., ~5.0–5.8, signifying that in those species, protein will apparently be precipitated in acidic buffer. This fact will be helpful in isoelectric focussing during purification of recombinant SH2 proteins by improvement of buffer systems. In longer proteins, presence of more charged amino acids is useful in buffering the effect of variations in their composition and can keep neutral pI close to 7.4. Wide variation in the pI of the SH2 protein than their size (range: 45653.59–68833.88 g/mol) synchronized with the report that the molecular weight of orthologous proteins was much more conserved than their isoelectric point [60].

Computation of Instability index (Ii) helped in predicting *in vivo* half-life (T_1/2_) of *SH2* protein in different species. Previously, it was reported that proteins with Ii> 40 have a T_1/2_ of less than 5h, whereas those with <40 have a longer T_1/2_ of 16h [61]. In our study, SH2 protein in 22 genotypes out of 37 was stable and in others, it was thermally unstable. GRAVY indices of SH2 sequences ranged from -0.259 to -0.015 reflecting the hydrophobic nature of the amino acids [62]. AGPases of cereal crops are readily denatured at high temperatures. AGPase of maize endosperm loses 74–96% of its activity upon heating at 57–60°C for 5 min [63]. This could be the one of the possible reasons of better heat tolerance ability in maize compared to many other cereals like wheat [64]. Aliphatic index is another important criterion which measures relative volume occupied by the aliphatic side chains of the amino acids such as alanine, leucine, isoleucine and valine, and also serves as a measure of thermostability of proteins [65]. Lu et al. [66] compared 110 pairs of homologous mesophilic and thermophilic proteins for their amino acid composition and found that due to more leucine residues, thermophilic proteins have higher value of GRAVY and aliphatic index. Similar to earlier reports, there was frequent occurrence and conservation of leucine residues in case of SH2 protein in maize as well as in orthologues. Higher aliphatic index implies that an increased thermostability of the proteins may support an increase in their solubility and again emphasizes the conclusion that thermostability and solubility are correlated positively with each other. Among the *sh2* mutant and wild genotypes of maize, value of AI showed its thermostability over a wide range of temperatures. On the other hand, when SH2 orthologues were compared with each other, the accession of *O*. *sativa* var. *indica* (B8AQH10), was found to have maximum AI (101.24), followed by the orthologue in *S*. *italica* (K4AAH8:100.96) and *O*. *sativa* var. *japonica* (Q10Q61: 100.51), depicting their very high thermostability and hence soluble nature.

### Phylogenetic relationship of *sh2* gene and large subunit of AGPase among maize and orthologues

DNA and protein sequence variations among different orthologues provide precise information on the divergence of alleles in various crops that can be exploited in phylogenetic and evolutionary studies. Among the eight monocots studied in the present investigation, one of the orthologues in *A*. *tauschii* with six exons, had two longer introns in it, while the second orthologue, had 13 exons with intermediate size of introns. However, *T*. *aestivum* had the maximum number of exons (21) in one of the orthologues with smaller length of introns [67]. In an investigation on comparative analysis of AGPases with special emphasis on wheat carried out by Batra et al. [68], it was suggested that during evolution, introns of large subunit genes got divided and resulted in smaller introns in monocots. Introns in orthologues of *S*. *italica* and *S*. *bicolor* were larger in length as intron-12 in case of maize. Long introns are generally favoured as they intensify the natural selection efficiency by releasing Hill-Robertson (HR) effect. This showed that introns might be involved in decreasing intragenic HR effect between locations which are under the impact of natural selection in finite populations. In a gene, a positive correlation was reported between the intronic burden and its evolutionary conservation [69] and the negative correlation was observed for gene expression with the total size of introns and number of introns. Genes possessing higher intronic burden had smaller density of nonsense and missense mutations in coding regions, suggesting that genes are under stronger pressure from purifying selection. Further, the clustering pattern in the present study revealed that maize *sh2* gene is closer to rice *sh2* orthologues compared to other monocots. This indicated that *sh2* gene of rice and wheat diverged much later as compared to earlier divergence of *sh2* in wheat, sorghum, barley, foxtail millets, *Brachypodium* and *Aegilops*.

The domain study in protein sequences revealed that the AGPase domain is more conserved than the NTP-transferase domain. Less polymorphism viz., only 0–5 amino acid substitutions in the inbreds viz., SH2*-*mutant2 (5) >SH2-Wild4 (4) >SH2*-*mutant3 (3) >SH2-Wild2 (2) >SH2*-*mutant1 (1) >SH2*-*mutant4 (0), might be due to positive selection pressure. Generally, wild ancestors show more variations than their domesticated descendants. It might be during the course of evolution when some of the cereal crops like wheat and barley stopped responding to the effectors, 3-PGA and P_i_, whereas maize and rice retained this mechanism of allosteric regulation. The AGPase in rice endosperm aligns closer to that of maize in terms of allosteric regulation. Since the primary sequences of small subunit of AGPase in different species are more conserved, the differences in allosterically regulated properties are perhaps due to the differences in large subunit. In a study on rice AGPases conducted by Tuncel et al. [49], alignment of the primary sequences of larger subunit showed that it shared 77% identity with that of maize, while it shared lesser identity of 71% with large subunit of wheat and barley. The phylogenetic trees generated from nucleotide and protein sequence data were shown to be consistent in cluster formation, and SH2 protein of the maize indicated the conservation of AGPase domain. The evolutionary analyses of SH2 protein in maize revealed that the division of cluster-A1 corresponded well with domain structures and sequence conservation. The reason behind the SH2 proteins of other monocots being available in different cluster could be due to similar functional relationship.

The present investigation studied allelic variation in *sh2* gene among 48 exotic- and indigenous- maize inbreds of sweet- and field- corn types. The study revealed that diverse allelic haplotypes of *sh2* gene were present in sweet corn and field corn inbreds. The study also provided details of gene architecture and evolutionary linkages in *sh2* gene, and physicochemical properties and secondary structure of large sub-unit of AGPase protein encoded by *sh2*. This study reports detailed characterization and allelic diversity analysis of *sh2* gene in a set of diverse maize inbreds and various orthologues in related monocots.

## Supporting information

S1 Table Pedigree details of *Sh2* mutant and *Sh2*-wild genotypes used in present study. (DOCX)

S2 Table Source institute of 48 maize inbreds. (DOCX)

S3 Table List of selected *sh2* inbreds, wild inbreds and orthologues of *Sh2* gene in monocots. (DOCX)

S4 Table Evolutionary distance and synonymous and non-synonymous scores in comparison with reference to Sh2-Wild-M81603 allele. (DOCX)

S5 Table Variations identified in AGPase and NTP transferase domain in selected maize genotypes. (DOCX)

S1 Fig Evolutionary relationship of SH2 protein in maize and its orthologues in selected monocots. (TIF)

S2 Fig Domains in SH2 protein; (a) in SH2-AAB52952.1 protein and (b) Comparison of SH2 protein mutants and wild inbreds. (TIF)

S3 Fig (a) Secondary structure prediction and (b) FoldIndex and disorder in SH2 protein. (TIF)
