# Supplementary material for: Allelic variation in shrunken2 gene affecting kernel sweetness in exotic-and indigenous-maize inbreds
Source: PLoS One. 2022 Sep 22;17(9):e0274732. doi: 10.1371/journal.pone.0274732 (PMC9498942; doi:10.1371/journal.pone.0274732)
Supplement: S1 Table — (DOCX) [file pone.0274732.s001.docx]

**S1 Table** Details of *Sh2* mutant and *Sh2*-wild genotypes used for sequencing of entire *sh2* gene

| **S. No.** | **Inbred** | **Code** | **Source** |
| --- | --- | --- | --- |
| 1 | HKI-1348 | *Sh2*-Wild1 | ICAR-IARI, New Delhi |
| 2 | HKI-1105 | *Sh2*-Wild2 | ICAR-IARI, New Delhi |
| 3 | CML-425 | *Sh2*-Wild3 | ICAR-IARI, New Delhi |
| 4 | LM-13 | *Sh2*-Wild4 | CCS-HAU, Uchani |
| 5 | UMI-1200 | *Sh2*-Wild5 | ICAR-VPKAS, Almora |
| 6 | PMI-SH1 | *sh2*-Mutant1 | ICAR-IARI, New Delhi |
| 7 | PMI-SH2 | *sh2*-Mutant2 | ICAR-IARI, New Delhi |
| 8 | PMI-SH3 | *sh2*-Mutant3 | ICAR-IARI, New Delhi |
| 9 | PMI-SH4 | *sh2*-Mutant4 | ICAR-IARI, New Delhi |
| 10 | PMI-SH5 | *sh2*-Mutant5 | ICAR-IARI, New Delhi |
| 11 | PMI-SH6 | *sh2*-Mutant6 | ICAR-IARI, New Delhi |
