# Supplementary material for: Allelic variation in shrunken2 gene affecting kernel sweetness in exotic-and indigenous-maize inbreds
Source: PLoS One. 2022 Sep 22;17(9):e0274732. doi: 10.1371/journal.pone.0274732 (PMC9498942; doi:10.1371/journal.pone.0274732)
Supplement: S2 Table — (DOCX) [file pone.0274732.s002.docx]

**S2 Table** Details of 48 maize inbreds used for gene-based diversity analysis employing using *InDel*-based markers

| **S. No.** | **Inbreds** | **Wild/ Mutant** | **Source** |
| --- | --- | --- | --- |
| 1 | PMI-SH7 | Mutant (*sh2sh2*) | ICAR-IARI, New Delhi |
| 2 | PMI-SH8 | Mutant (*sh2sh2*) | ICAR-IARI, New Delhi |
| 3 | PMI-SH9 | Mutant (*sh2sh2*) | ICAR-IARI, New Delhi |
| 4 | PMI-SH11 | Mutant (*sh2sh2*) | ICAR-IARI, New Delhi |
| 5 | PMI-SH14 | Mutant (*sh2sh2*) | ICAR-IARI, New Delhi |
| 6 | PMI-SH19 | Mutant (*sh2sh2*) | ICAR-IARI, New Delhi |
| 7 | PMI-SH21 | Mutant (*sh2sh2*) | ICAR-IARI, New Delhi |
| 8 | PMI-SH24 | Mutant (*sh2sh2*) | ICAR-IARI, New Delhi |
| 9 | PMI-SH26 | Mutant (*sh2sh2*) | ICAR-IARI, New Delhi |
| 10 | PMI-SH27 | Mutant (*sh2sh2*) | ICAR-IARI, New Delhi |
| 11 | PMI-SH33 | Mutant (*sh2sh2*) | ICAR-IARI, New Delhi |
| 12 | PMI-SH44 | Mutant (*sh2sh2*) | ICAR-IARI, New Delhi |
| 13 | PMI-SH46 | Mutant (*sh2sh2*) | ICAR-IARI, New Delhi |
| 14 | PMISH48 | Mutant (*sh2sh2*) | ICAR-IARI, New Delhi |
| 15 | PMI-SH50 | Mutant (*sh2sh2*) | ICAR-IARI, New Delhi |
| 16 | PMI-SH51 | Mutant (*sh2sh2*) | ICAR-IARI, New Delhi |
| 17 | PMI-SH57 | Mutant (*sh2sh2*) | ICAR-IARI, New Delhi |
| 18 | PMI-SH58 | Mutant (*sh2sh2*) | ICAR-IARI, New Delhi |
| 19 | PMI-SH66 | Mutant (*sh2sh2*) | ICAR-IARI, New Delhi |
| 20 | PMI-SH70 | Mutant (*sh2sh2*) | ICAR-IARI, New Delhi |
| 21 | PMI-SH71 | Mutant (*sh2sh2*) | ICAR-IARI, New Delhi |
| 22 | PMI-SH75 | Mutant (*sh2sh2*) | ICAR-IARI, New Delhi |
| 23 | PMI-SH80 | Mutant (*sh2sh2*) | ICAR-IARI, New Delhi |
| 24 | HKI-323 | Wild (*Sh2Sh2*) | CCS-HAU, Uchani, Karnal |
| 25 | HKI-1105 | Wild (*Sh2Sh2*) | CCS-HAU, Uchani, Karnal |
| 26 | HKI-1128 | Wild (*Sh2Sh2*) | CCS-HAU, Uchani, Karnal |
| 27 | HKI-161 | Wild (*Sh2Sh2*) | CCS-HAU, Uchani, Karnal |
| 28 | HKI-163 | Wild (*Sh2Sh2*) | CCS-HAU, Uchani, Karnal |
| 29 | HKI-193-1 | Wild (*Sh2Sh2*) | CCS-HAU, Uchani, Karnal |
| 30 | HKI-193-2 | Wild (*Sh2Sh2*) | CCS-HAU, Uchani, Karnal |
| 31 | PMI-PV3 | Wild (*Sh2Sh2*) | ICAR-IARI, New Delhi |
| 32 | UMI-1200 | Wild (*Sh2Sh2*) | TNAU, Coimbatore |
| 33 | UMI-1230 | Wild (*Sh2Sh2*) | TNAU, Coimbatore |
| 34 | BML-6 | Wild (*Sh2Sh2*) | PJTSAU, Telangana S |
| 35 | BML-7 | Wild (*Sh2Sh2*) | PJTSAU, Telangana |
| 36 | LM-13 | Wild (*Sh2Sh2*) | PAU, Ludhiana |
| 37 | LM-14 | Wild (*Sh2Sh2*) | PAU, Ludhiana |
| 38 | CML-40 | Wild (*Sh2Sh2*) | CIMMYT, Mexico |
| 39 | CML-49 | Wild (*Sh2Sh2*) | CIMMYT, Mexico |
| 40 | CML-52 | Wild (*Sh2Sh2*) | CIMMYT, Mexico |
| 41 | CML-202 | Wild (*Sh2Sh2*) | CIMMYT, Mexico |
| 42 | CML-226 | Wild (*Sh2Sh2*) | CIMMYT, Mexico |
| 43 | CML-235 | Wild (*Sh2Sh2*) | CIMMYT, Mexico |
| 44 | CML-254 | Wild (*Sh2Sh2*) | CIMMYT, Mexico |
| 45 | CML-269 | Wild (*Sh2Sh2*) | CIMMYT, Mexico |
| 46 | CML-321 | Wild (*Sh2Sh2*) | CIMMYT, Mexico |
| 47 | CML-383 | Wild (*Sh2Sh2*) | CIMMYT, Mexico |
| 48 | VQL-1 | Wild (*Sh2Sh2*) | ICAR-VPKAS, Almora |
