# Supplementary material for: Allelic variation in shrunken2 gene affecting kernel sweetness in exotic-and indigenous-maize inbreds
Source: PLoS One. 2022 Sep 22;17(9):e0274732. doi: 10.1371/journal.pone.0274732 (PMC9498942; doi:10.1371/journal.pone.0274732)
Supplement: S3 Table — (DOCX) [file pone.0274732.s003.docx]

**S3 Table** List of selected *sh2* inbreds, wild inbreds and orthologues of *Sh2* gene in monocots

| **S. No.** | ***Sh2* sequence** | **Gene ID** | **Protein ID** |
| --- | --- | --- | --- |
| 1 | *Z. mays* (s*h2*-Mutant1) | Nucleotide sequence generated in the present study | Protein sequence translated using generated nucleotide sequence |
| 2 | *Z. mays* (*sh2*-Mutant2) |  |  |
| 3 | *Z. mays*(*sh2*-Mutant3) |  |  |
| 4 | *Z. mays* (*sh2*-Mutant4) |  |  |
| 5 | *Z. mays* (*sh2*-Mutant5) |  |  |
| 6 | *Z. mays* (*sh2*-Mutant6) |  |  |
| 7 | *Z. mays* (*Sh2*-Wild1) |  |  |
| 8 | *Z. mays* (*Sh2*-Wild2) |  |  |
| 9 | *Z. mays* (*Sh2*-Wild3) |  |  |
| 10 | *Z. mays* (*Sh2*-Wild4) |  |  |
| 11 | *Z. mays* (*Sh2*-Wild5) |  |  |
| 12 | *Z. mays* (*Sh2*-Wild-M81603) | M81603 | AAB52952.1 |
| 13 | *O. sativa*var. japonica | NC_029260.1 | Q688T8 |
| 14 |  | NC_029256.1 | Q7G065 |
| 15 |  | NC_029262.1 | Q0D7I3 |
| 16 |  | NC_029258.1 | Q10Q61 |
| 17 |  | NC_029258.1 | Q6AVT2 |
| 18 | *B. distachyon* | NC_016131.3 | I1H8B7 |
| 19 |  | NC_016132.3 | I1HFZ1 |
| 20 | *H. vulgare* | Chromosome 4H:  569,157,417-569, 162, 970 | A0A287PI95 |
| 21 |  | Chromosome 1H:  548,765,935-548, 770, 616 | C3W8L1 |
| 22 | *S. bicolor* | NC_012870.2 | C5WTQ1 |
| 23 |  | NC_012870.2 | C5WLV9 |
| 24 |  | NC_012878.2 | A0A1Z5R3X9 |
| 25 | *O. sativa*var. indica | Chromosome 3:  34,318,562-34,322,802 | B8AR31 |
| 26 |  | Chromosome 5:  30,219,471-30,222,858 | A2Y7W1 |
| 27 |  | Chromosome 1:  28,487,624-28,491,651 | P93430 |
| 28 |  | Chromosome 3:  6,154,994-6,158,995 | B8AQH0 |
| 29 | *S. italica* | NC_028458.1 | K4AAH8 |
| 30 |  | NC_028458 | K4A7B2 |
| 31 |  | NC_028452.1 | K3Z5F3 |
| 32 | *T. aestivum* | Chromosome 5B:  656,228,370-656,233,066 | A5GZ74 |
| 33 |  | Chromosome 1B:  668,128,722-668,133,731 | Q7XJA9 |
| 34 |  | Chromosome 1D:  479,889,259-479,894,164 | P12299 |
| 35 | *A. tauschii* | NW_017913381.1 | M8AY46 |
| 36 |  | Chromosome 1D:  487,587,537-487,592,109 | A0A0U4H004 |
| 37 |  | NW_017912774.1 | M8BCF1 |
