# Supplementary material for: Allelic variation in shrunken2 gene affecting kernel sweetness in exotic-and indigenous-maize inbreds
Source: PLoS One. 2022 Sep 22;17(9):e0274732. doi: 10.1371/journal.pone.0274732 (PMC9498942; doi:10.1371/journal.pone.0274732)
Supplement: S4 Table — (DOCX) [file pone.0274732.s004.docx]

**S4 Table** Evolutionary distance and synonymous and non-synonymous scores in comparison with reference *Sh2* allele

| **S. No.** | **Genotype** | **Pairwise distance** | **Ks** | **Ka** | **Ka/Ks** |
| --- | --- | --- | --- | --- | --- |
| 1 | *sh2*-Mutant1 | 0.013 | 0.0266 | 0.0073 | 0.274 |
| 2 | *sh2*-Mutant2 | 0.018 | 0.0245 | 0.0128 | 0.522 |
| 3 | *sh2*-Mutant3 | 0.019 | 0.0305 | 0.0128 | 0.419 |
| 4 | *sh2*-Mutant4 | 0.010 | 0.0206 | 0.0057 | 0.276 |
| 5 | *sh2*-Mutant5 | 0.019 | 0.0245 | 0.0144 | 0.587 |
| 6 | *sh2*-Mutant6 | 0.162 | 0.1717 | 0.1487 | 0.866 |
| 7 | *Sh2*-Wild1 | 0.017 | 0.0176 | 0.0130 | 0.738 |
| 8 | *Sh2*-Wild2 | 0.017 | 0.0176 | 0.0114 | 0.647 |
| 9 | *Sh2*-Wild3 | 0.016 | 0.0087 | 0.0122 | 1.402 |
| 10 | *Sh2*-Wild4 | 0.019 | 0.0176 | 0.0163 | 0.926 |
| 11 | *Sh2*-Wild5 | 0.053 | 0.0602 | 0.0449 | 0.745 |
