# Supplementary material for: Allelic variation in shrunken2 gene affecting kernel sweetness in exotic-and indigenous-maize inbreds
Source: PLoS One. 2022 Sep 22;17(9):e0274732. doi: 10.1371/journal.pone.0274732 (PMC9498942; doi:10.1371/journal.pone.0274732)
Supplement: S5 Table — (DOCX) [file pone.0274732.s005.docx]

**S5 Table** Variations identified in AGPase and NTP transferase domain in selected maize genotypes

| **S. No.** | **Genotypes** | **AGPase domain** | **NTP transferase domain** | | |
| --- | --- | --- | --- | --- | --- |
|  |  | **Deletion (aa*)** | **Insertion**  **(aa*)** | **Deletion**  **(aa*)** | **Substitution (aa*)** |
| 1 | *Sh2*-Mutant1 | - | - | - | 1 |
| 2 | *Sh2*-Mutant2 | - | - | - | 6 |
| 3 | *Sh2*-Mutant3 | - | - | - | 3 |
| 4 | *Sh2*-Mutant4 | - | - | - | 0 |
| 5 | *Sh2*-Mutant5 | - | - | 26 | 2 |
| 6 | *Sh2*-Mutant6 | 295-305 | - | 74 | 1 |
| 7 | *Sh2*-Wild1 | - | - | - | 2 |
| 8 | *Sh2*-Wild2 | 90-109 | - | 80 | 2 |
| 9 | *Sh2*-Wild3 | - | - | - | 4 |
| 10 | *Sh2*-Wild4 | 90-109 | - | 10, 54 | 14 |
| 11 | *Sh2*-Wild5 | 179-187 | 2, 12 | 110, 6 | 11 |

*aa: amino acids
