# Supplementary figures and images for: Allelic variation in shrunken2 gene affecting kernel sweetness in exotic-and indigenous-maize inbreds
Source: PLoS One. 2022 Sep 22;17(9):e0274732. doi: 10.1371/journal.pone.0274732 (PMC9498942; doi:10.1371/journal.pone.0274732)

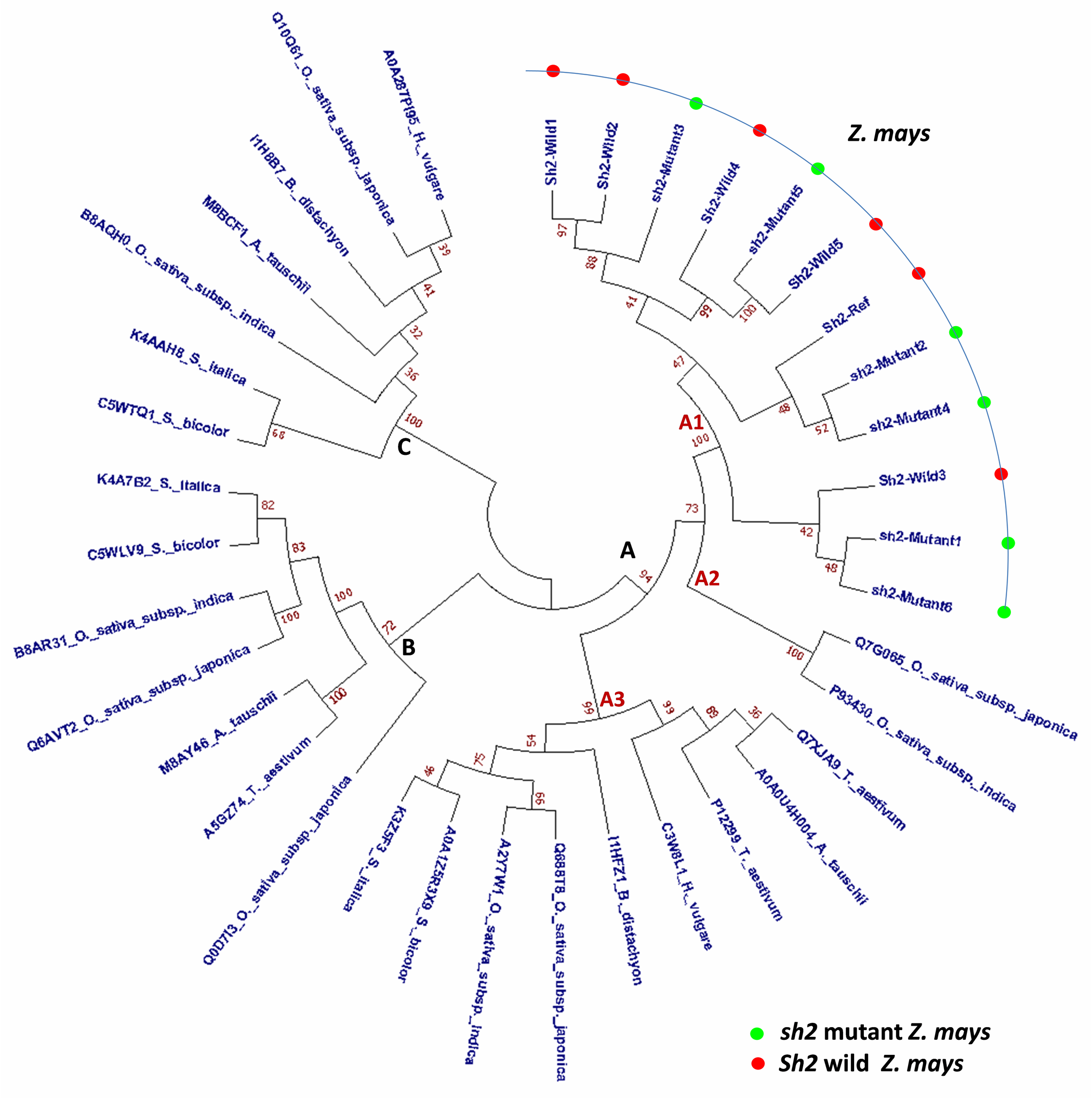

Supplement: S1 Fig — (TIF) [file pone.0274732.s006.tif]

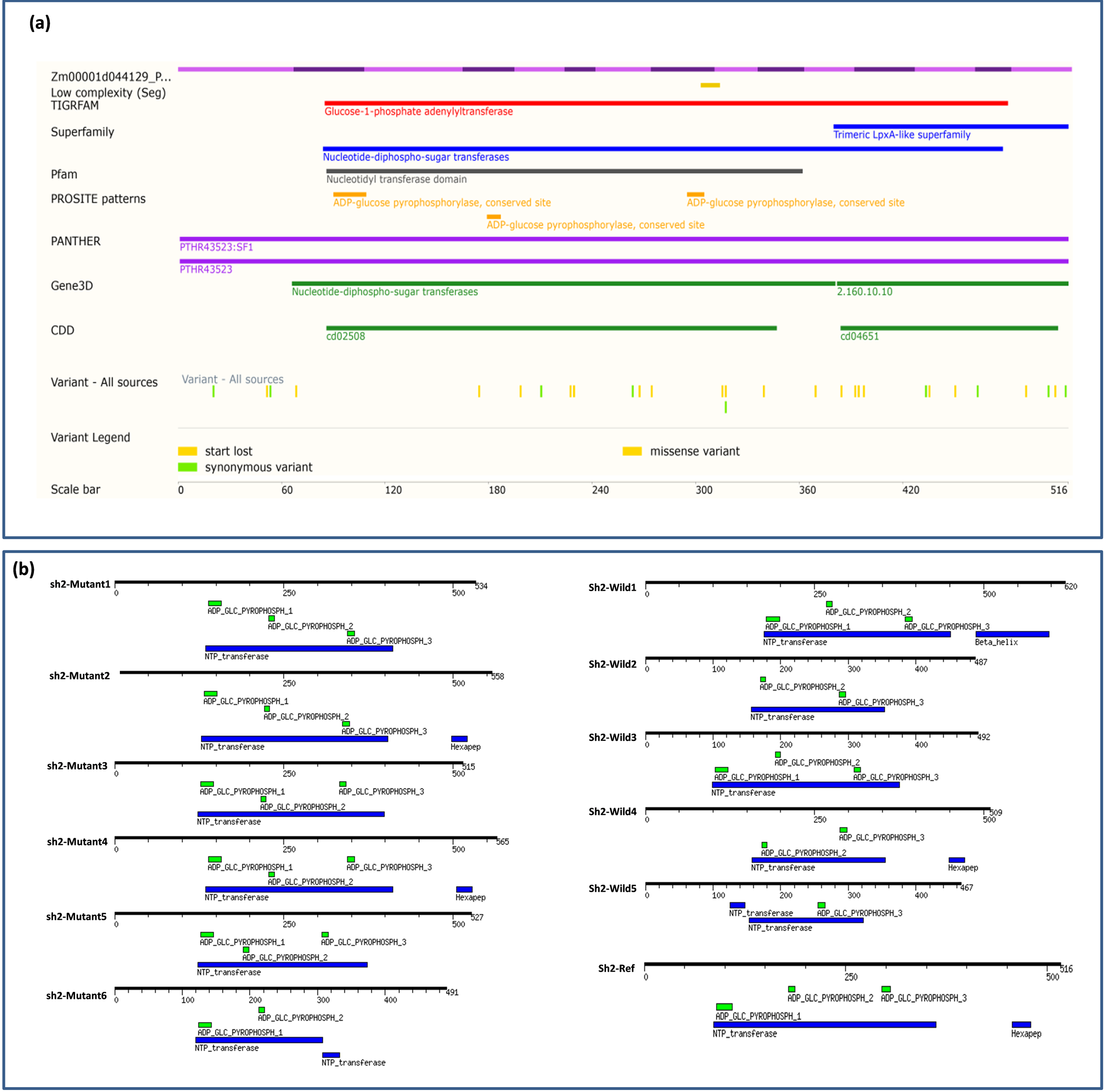

Supplement: S2 Fig — (TIF) [file pone.0274732.s007.tif]

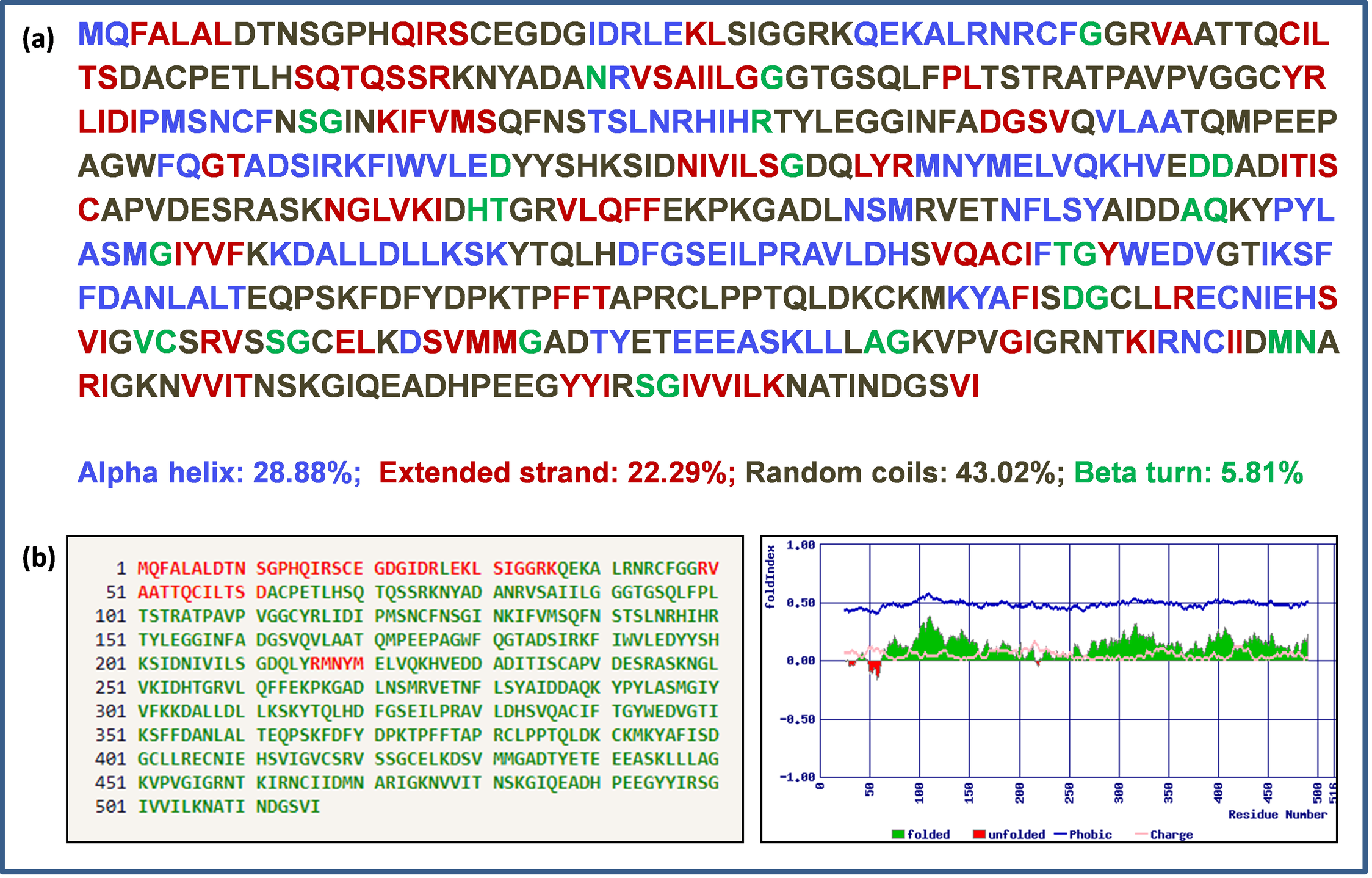

Supplement: S3 Fig — (TIF) [file pone.0274732.s008.tif]
